# The effect of soy isoflavones supplementation on metabolic status in patients with non-alcoholic fatty liver disease: a randomized placebo controlled clinical trial

**DOI:** 10.1186/s12889-024-18812-3

**Published:** 2024-05-21

**Authors:** Asal Neshatbini Tehrani, Behzad Hatami, Ghazal Daftari, Azita Hekmatdoost, Zahra Yari, Amin Salehpour, Seyed Ahmad Hosseini, Bizhan Helli

**Affiliations:** 1 Student Research Committee, Ahvaz Jundishapur University of Medical Sciences, Ahvaz, Iran; 2 Department of Nutrition, School of Allied Medical Sciences, Ahvaz Jundishapur University of Medical Sciences, Golestan Boulevard, Ahvaz, 78531-67465 Iran; 3 Nutrition and Metabolic Diseases Research Center, Clinical Research Institute, Ahvaz Jundishapur University of Medical Sciences, Ahvaz, Iran; 4 Gastroenterology and Liver Diseases Research Center, Research Institute for Gastroenterology and Liver Diseases, Shahid Beheshti University of Medical Sciences, Tehran, Iran; 5 School of Medicine, Tehran University of Medical Sciences, Tehran, Iran; 6 Clinical Nutrition and Dietetics Department, Faculty of Nutrition Sciences and Food Technology, National Nutrition and Food Technology Research Institute, Shahid Beheshti University of Medical Sciences, Tehran, Iran; 7 Department of Nutrition Research, National Nutrition and Food Technology Research Institute, Faculty of Nutrition Sciences and Food Technology, Shahid Beheshti University of Medical Sciences, Tehran, Iran; 8 School of Public Health, Occupational Health Research Center, Iran Universityof Medical Sciences, Tehran, Iran

**Keywords:** Non-alcoholic fatty liver disease, Isoflavone, Soy, Metabolic factors

## Abstract

**Background:**

Non-alcoholic fatty liver disease (NAFLD) accounts as a crucial health concern with a huge burden on health and economic systems. The aim of this study is to evaluate the effect of soy isoflavones supplementation on metabolic status in patients with NAFLD.

**Methods:**

In this randomized clinical trial, 50 patients with NAFLD were randomly allocated to either soy isoflavone or placebo groups for 12 weeks. The soy isoflavone group took 100 mg/d soy isoflavone and the placebo group took the similar tablets containing starch. Anthropometric indices, blood lipids, glycemic parameters and blood pressure were measured at the beginning and at the end of the study.

**Results:**

At the end of week 12 the level of serum triglyceride (TG), low density lipoprotein (LDL) and total cholesterol (TC) was significantly decreased only in soy isoflavone group compared to baseline (*P* < 0.05). Although waist circumference (WC) decreased significantly in both groups after 12 weeks of intervention (*P* < 0.05), hip circumference (HC) decreased significantly only in soy isoflavone group (*P* = 0.001). No significant changes observed regarding high density lipoprotein (HDL) and blood pressure in both groups. At the end of the study, serum glucose level was significantly decreased in the placebo group compared to baseline (*P* = 0.047). No significant changes demonstrated in the soy isoflavone group in regard to glycemic parameters (*P* > 0.05).

**Conclusions:**

This study revealed that soy isoflavones could significantly reduce TG, LDL TC, WC and HC in NAFLD patients.

**Trial registration:**

The Ethics committee of Ahvaz Jundishapur University of Medical Sciences approved the protocol of the present clinical research (**IR.AJUMS.REC.1401.155**). The study was in accordance with the Declaration of Helsinki. This study’s registered number and date are **IRCT20220801055597N1** and **20.09.2022**, respectively at https://fa.irct.ir.

## Background

Non-alcoholic fatty liver disease (NAFLD) is considered as a main public health worldwide. The major culprit for causing NAFLD is fat accumulation in hepatocytes so that it occupies more than 5% of the weight or volume of the liver. If left untreated, this disease is gradually developing to more severe types and leading to fibrosis and cirrhosis [1]. A relatively recent estimation has reported that the prevalence of NAFLD is approximately 20–30% in general population [2]. Also, a high prevalence of this disease is observed among Iranian people who are living in a developing country [3]. There are several risk factors attributed to NAFLD including components of metabolic syndrome like dyslipidemia, diabetes type 2 and increasing the body mass index (BMI) which, the two latter are considered as crucial factors for NAFLD development because two-thirds of people suffering from obesity and diabetes are diagnosed with hepatic steatosis [4, 5]. Genetic propensity and environmental factors such as unhealthy dietary pattern and inactive lifestyle compromise the multifactorial nature of the NAFLD pathogenesis [6, 7]. A new terminology named metabolic associated fatty liver disease (MAFLD) has been introduced recently. Presence of liver steatosis along with at least one of the criteria related to metabolic syndrome will lead to categorize patients into MAFLD [8, 9]. Improving diet and physical activity levels are the main strategy for most of the patients with NAFLD, nonetheless, the adherence rate is low and pharmacological therapy remains useless in these patients [10]. According to several previous studies [11–13], some dietary supplements with anti-inflammatory, insulin sensitizing and antioxidant activities could reinforce the impact of lifestyle modification in NAFLD treatment. Recently, a huge attention has been paid to isoflavones due to their positive effects on cardiovascular related risk factors [14, 15]. Although a review study showed the null effect of soy protein and isoflavones on HDL-C, triglyceride, blood pressure and lipoprotein(a) [16], some other studies indicated that isoflavones or food sources of that such as soy can decrease the level of serum total cholesterol, LDL-C, triglyceride and increase the level of HDL-C [17–19]. Isoflavones as ligands for some proteins with lipid regulation role such as peroxisome proliferator activated receptors (PPARs) can decrease the synthesis of liver lipids and bile acids and decline the reabsorption of cholesterol [20], thus, inserting their lipid lowering effects [20]. There are also some studies indicating the positive effects of soy isoflavones on blood pressure in patients with NAFLD [21, 22]. The mechanisms involved are targeting the genes in charge of production of vasodilation agents such as nitric oxide (NO) [23]. Moreover, previous studies have suggested the role of soy isoflavones on improving the glycemic status [24, 25]. some of the underlying mechanisms including soy isoflavone’s effects on inhibition of intestinal glucose transport [26] and tyrosine kinase activity [27], α glucosidase alterations in number and affinity of insulin receptors [28] and activation of PPARs [29].

The aim of the present paper was to investigate the effect of soy isoflavones supplementation on metabolic status in management of NAFLD.

## Methods

### Study design and participants recruitment

Sample size was estimated based on Fibroscan controlled attenuation parameter (CAP) score with consideration of 10 unit alteration in the mean CAP score with a power of 80% (β = 20%). The minimum sample size determined as 21 patients in each group. Finally, due to loss of patients, 25 subjects were enrolled [30]. Sample size was calculated according to it’s formula [31].

**Fig. 1:**
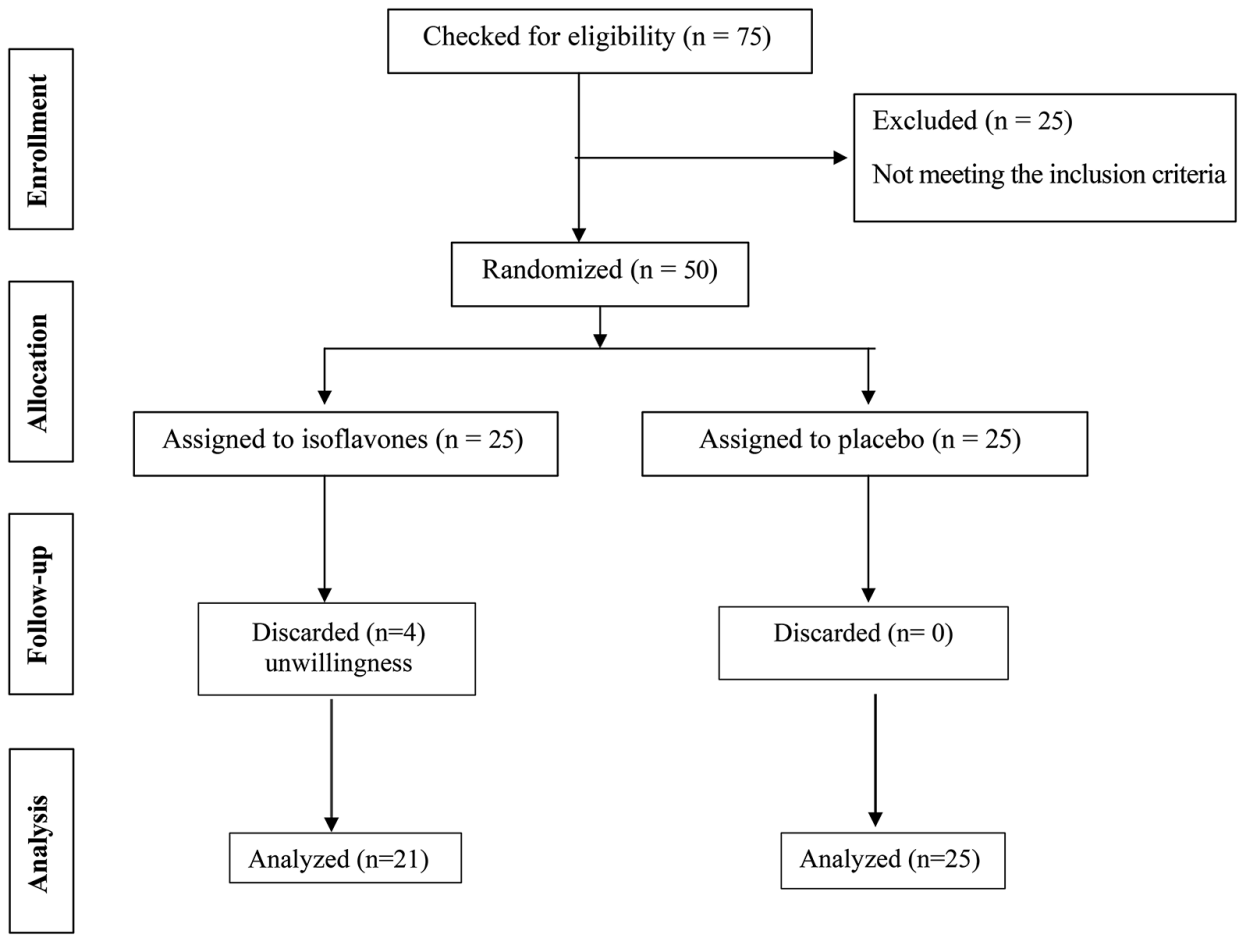
Study flow chart

In this placebo-controlled, double-blind randomized clinical trial, a total of 75 patients with NAFLD were enrolled to assess the inclusion criteria; of these, 50 people were eligible to participate, of which four refused to continue cooperation. Therefore, 46 patients completed the study (Fig. 1). The participants were recruited from Taleghani Hospital Hepatology Clinic (THHC) with a diagnosis of NAFLD based on Fibroscan examination. In the present study, the data were collected from September 2022 to May 2023. The inclusion criteria were [1] 18 years or older; [2] without any history of allergy to soy or having the soy, soy products and/or soy supplements in massive amount or as a dietary habit [3], without chronic diseases including renal, liver, heart, respiratory, cardiovascular, malignancies, auto immune disorders, cushing’s syndrome, thyroid dysfunction, hepatitis, cirrhosis, biliary disorders, diabetes, gastrointestinal tract diseases affecting the gut absorption and psychiatric disorders considering as an obstacle for patients to prepare written informed consent; [4] hepatic steatosis grade 2 and higher with fibroscan confirmation (CAP > 260 dB/m); [5] without history of excessive alcohol drink (≥ 10 g/day); [6] without history of drug consumption with approved positive effects on NAFLD treatment (i.e. metformin, vitamin E, ursodeoxycholic acid, phenytoin, tamoxifen, lithium, corticosteroids and methotrexate) in last three months; [7] without the history of bariatric surgery or following weight loss diets within 6 months; [8] without history of smoking; [9] not being a pregnant or lactating woman; [10] not following the particular diets within the last 6 months.

### Intervention

Participants were randomly allocated to either a soy isoflavone or placebo group by block randomization based on gender and menopause status. Then by a random number table, the subjects were assigned to take either soy isoflavones or placebo for 12 weeks. Patients in soy isoflavone group received 100 mg of soy isoflavones as two tablets per day for 12 weeks. Each tablet contained 50 mg soy isoflavones with combination of 31.86 mg of genistin, 1.49 mg of genistein, 13.21 mg of daidzin, 1.75 mg of daidzein, 1.14 mg of glycitin, and 0.55 mg of glycitein. There is no side effects regarding the consumption of 100 mg/day soy isoflavones [32–34]. The participants in placebo group received two tablets of placebo filled with starch. The placebo tablets were similar to soy isoflavone tablets regarding the appearance, taste, odor and bottles by which supplying to subjects. Both placebo and soy isoflavone tablets were produced by Gol Daru Pharmaceutical Company, Esfahan, Iran. Then, tablets were labeled as A or B by a third party, in this way both patients and researchers were blinded to the sample selection as well as the type of intervention.

Patients were followed up every four weeks at weeks 4, 8 and 12 from the study initiation. Baseline characteristics of participants were obtained at the first visit (week 0) and they were given a 4-week supply of soy isoflavone or placebo tablets. At weeks 4 and 8, the participants were again provided with enough supply of tablets. In the present study, we measured the compliance rate to study protocol by controlling the number of remaining tablets in each visit. Patients who had not used the minimum of 90% of the given tablets were excluded from the study. The advice on lifestyle modification was given to both groups including a gradual increase in physical activity (tree times per week for at least 30 min of middle intensity physical activity) and nutrition counseling based on clinical guidelines of National Institute of Health and the North American Association for the Study of Obesity [35]. According to the mentioned guidelines, the patients were advised to reduce the daily calories intake, consume fat-modified foods, limit the intake of saturated fats and cholesterol to less than 7% of total calories and 200 mg/day, respectively, include more proteins from plant sources, eat more complex carbohydrates from vegetables, fruits, whole grains, etc., follow a diet rich in fiber and intake adequate vitamins and minerals along with persistent moderate levels of physical activity.

### Clinical and para-clinical assessments

We measured anthropometric parameters (weight, waist and hip circumferences) at the beginning (week 0) and at the end of the study (week 12). Height was measured at the beginning of the study using a portable non-stretch meter with bare feet and it was recorded to the nearest of 0.5 cm. weight was measured using a Squeal Scale to an accuracy of 100 g. waist and hip circumferences were measured by a non-stretch meter at the narrowest part of the waist and the widest part of the hip, respectively. BMI was calculated as weight in kilograms dividing by height in squared meters. Waist to hip ratio (WHR) measured by dividing the waist circumference to hip circumference both in centimeters. In order to assess the dietary intakes of patients we used a 3-day dietary recalls (two weekdays and one weekend) at the beginning of the study and at the end of the week 12. After gathering information on dietary record, all data was monitored by a dietitian in terms of completeness. Then, Nutritionist 4 software was applied to analyze dietary information (N Squared Computing, San Bruno, CA, USA). Subject’s physical activity level assessed by a semi-quantitative questionnaire at beginning and at the end of the study and it is described as metabolic equivalent hours per day (MET.h.d). The validity of this questionnaire was formerly checked among Iranian adolescents [36]. Systolic blood pressure (SBP) and diastolic blood pressure (DBP) were measured by a standard sphygmomanometer. The patients were advised to take a 15-minute rest and then the blood pressure was measured while they were sitting and the cuff was set on their right arm. This measurement was repeated twice with five minutes interval. We considered the average of two measurements in the analysis. In order to measure biochemical parameters, the subjects were advised to fast 12 h. Then, 7 ml of blood samples was taken from each participant. Blood samples remained at room temperature for 20 min, then centrifuge was done at 2,000 rpm for 10 min following the observation of clotting. Microtubes were used to separate serum and were frozen at − 80 °C until the time of analyze. In order to measure the level of serum triglyceride (TG), total cholesterol, low-density lipoprotein (LDL) and high-density lipoprotein (HDL) by enzymatic photometric method we used Delta Darman Part test kits (Delta darman part, Tehran, Iran). Fasting blood glucose (FBS) concentration was measured by enzymatic methods using Pars azmun kits (Parsazmun Co., Tehran, Iran). Serum insulin level assessed using ElectroChemiLuminescence (ECL) technology kits provided by Roche Company (Roche, Cobas, ECL, Germany). The following equation was used to calculate insulin resistance as homeostatic model assessment (HOMA-IR)= [fasting insulin (mU/L) × fasting blood glucose (mg/d)]/405 [37]. Quantitative insulin check index (QUICKI) was calculated according to its formula (1 / [log (FBS) + log (fasting insulin)]) as an indication for insulin sensitivity [38].

### Statistical analysis

The analysis of the present study’s data was conducted using the Statistical Package for the Social Sciences (SPSS, Inc., Chicago, IL, USA, version 21.0). To assess the normality of variables, Kolmogorov-Smirnov test was applied. The quantitative and categorical variables reported as mean ± SD and frequency (percentages), respectively. Regarding the comparison between groups at the beginning and at the end of the study the student’s unpaired t-test was used. To compare the mean changes within groups before and after supplementation, the student’s paired t-test was conducted. For considering the effectiveness of intervention in significant change of a variable, the analysis of covariance (ANCOVA) was performed. As confounding factors, the primary value of each parameter along with mean changes in BMI, WHR, energy intake and physical activity level were considered to make the ANCOVA’s model. The comparison of categorical variables conducted by a Chi-squared or Fisher’s exact test between the two groups. A *P* value of < 0.05 set as statistically significant.

## Results

According to participation flow chart (Fig. 1), four subjects assigned to placebo group discontinued the study due to unwillingness. The basic variables of the study participants in the placebo and soy isoflavone groups are illustrated in Table 1. As shown, there were no significant differences between the subjects in both groups regarding the baseline characteristics. Table 2 showed significant decline in WC in both groups (*P* < 0.05) and HC only in soy isoflavone group (*P* = 0.001) at the end of the study compared to pre-treatment. No significant changes reported in regard to other anthropometric indices. There were no significant changes in HDL-C in both groups (*P* > 0.05), while improvement in other lipid profile values including serum triglyceride, LDL-C and total cholesterol observed only in soy isoflavone group (*P* < 0.05) (Table 2). Serum glucose level was significantly decreased only in placebo group at the end of the study in compare with the study initiation (*P* = 0.047). Moreover, significant changes were reported in terms of QUICKI between the two groups at the end of the study (*P* = 0.014). Other glycemic variables remained insignificant (Table 2).

**Table 1:** Baseline characteristics of participants in the placebo and soy isoflavone groups^a^

variables	Placebo group (*n* = 21)	Soy isoflavone group (*n* = 25)	*P* value^b^
**Sex**			0.766
Male	10 (47.60)	10 (40.0)	
Female	11 (52.40)	15 (60.0)	
**Age**			
Male	46.0 ± 14.10	47.60 ± 14.98	0.809
Female	52.09 ± 5.73	51.93 ± 11.15	0.963
**Menopause status (females)**			1.00
Yes	5 (45.5)	6 (40.0)	
No	6 (54.5)	9 (60.0)	

^a^ Data are presented as mean ± SD for age and number (percentage) for sex and menopause status.

^b^Between group P value

**Table 2:** Mean changes (SD) from baseline in metabolic parameters by treatment groups

Metabolic parameters		Baseline	After 12 weeks	Changes (%)	*P* value^a^
**Anthropometric indices**					
Weight (kg)	Soy isoflavone group	84.0 ± 18.09	82.32 ± 17.23	-1.68 ± 5.91	0.166
	Placebo group	85.42 ± 14.34	82.66 ± 11.32	-2.76 ± 6.26	0.057
	*P* value ^b^	0.772	0.938	0.354^*c^	
WC (cm)	Soy isoflavone group	98.56 ± 11.99	94.86 ± 10.71	-3.69 ± 7.30	0.018
	Placebo group	97.19 ± 8.54	94.42 ± 7.09	-2.77 ± 4.20	0.007
	*P* value ^b^	0.664	0.871	0.888^*c^	
HC (cm)	Soy isoflavone group	112.88 ± 9.74	109.09 ± 8.96	-3.78 ± 5.14	0.001
	Placebo group	113.43 ± 10.06	109.55 ± 6.29	-3.87 ± 9.32	0.071
	*P* value ^b^	0.852	0.844	0.870^*c^	
BMI (kg/m^2^)	Soy isoflavone group	30.30 ± 4.91	28.38 ± 5.44	-1.2 ± 4.93	0.063
	Placebo group	29.95 ± 4.07	29.05 ± 3.13	-0.89 ± 2.86	0.167
	*P* value ^b^	0.790	0.617	0.438^*c^	
WHR	Soy isoflavone group	0.87 ± 0.05	0.86 ± 0.05	-0.003 ± 0.04	0.723
	Placebo group	0.861 ± 0.07	0.863 ± 0.07	0.002 ± 0.06	0.862
	*P* value ^b^	0.627	0.780	0.986^*c^	
**Blood pressure**					
SBP (mmHg)	Soy isoflavone group	113.60 ± 12.87	117.95 ± 12.49	4.35 ± 16.16	0.191
	Placebo group	120.95 ± 14.80	122.07 ± 10.08	1.12 ± 18.21	0.781
	*P* value ^b^	0.078	0.231	0.373^*c^	
DBP (mmHg)	Soy isoflavone group	77.0 ± 6.45	81.18 ± 10.77	4.17 ± 12.16	0.099
	Placebo group	83.33 ± 12.87	82.94 ± 9.86	-0.39 ± 16.58	0.915
	*P* value ^b^	0.036	0.568	0.524^*c^	
**Lipid profile**					
Triglyceride (mg/dl)	Soy isoflavone group	149.08 ± 60.19	105.39 ± 49.57	-43.68 ± 77.56	0.010
	Placebo group	161.14 ± 62.90	169.29 ± 91.13	8.15 ± 103.95	0.723
	*P* value ^b^	0.511	0.004	0.006^*c^	
HDL (mg/dl)	Soy isoflavone group	40.49 ± 9.51	42.42 ± 10.65	1.92 ± 12.83	0.461
	Placebo group	39.45 ± 908	39.22 ± 7.13	-0.22 ± 10.77	0.924
	*P* value ^b^	0.706	0.248	0.268^*c^	
LDL (mg/dl)	Soy isoflavone group	84.44 ± 19.42	68.15 ± 18.68	-16.28 ± 29.92	0.012
	Placebo group	96.95 ± 25.08	94.10 ± 31.90	-2.85 ± 25.40	0.613
	*P* value ^b^	0.06	0.001	0.010^*c^	
Total cholesterol (mg/dl)	Soy isoflavone group	117.84 ± 26.42	104.32 ± 26.43	-13.52 ± 19.79	0.002
	Placebo group	132.71 ± 28.85	138.30 ± 30.78	5.58 ± 21.61	0.250
	*P* value ^b^	0.075	≤ 0.001	0.003^*c^	
**Glycemic parameters**					
Glucose (mg/dl)	Soy isoflavone group	87.68 ± 17.73	82.68 ± 14.29	-4.99 ± 17.03	0.155
	Placebo group	107.0 ± 42.67	92.35 ± 23.44	-14.65 ± 31.74	0.047
	*P* value ^b^	0.045	0.093	0.753^*c^	
Insulin (mU/L)	Soy isoflavone group	25.61 ± 27.04	18.23 ± 18.37	-7.37 ± 30.60	0.240
	Placebo group	31.26 ± 34.55	18.09 ± 7.88	-13.16 ± 35.25	0.103
	*P* value ^b^	0.537	0.972	0.860^*c^	
HOMA-IR	Soy isoflavone group	5.92 ± 7.12	4.08 ± 4.52	-1.83 ± 7.84	0.252
	Placebo group	9.04 ± 11.21	4.14 ± 1.92	-4.89 ± 11.32	0.061
	*P* value ^b^	0.259	0.952	0.875^*c^	
QUICKI	Soy isoflavone group	0.32 ± 0.04	0.34 ± 0.05	0.02 ± 0.05	0.076
	Placebo group	0.30 ± 0.04	0.31 ± 0.02	0.01 ± 0.04	0.240
	*P* value ^b^	0.259	0.059	0.014^*c^	

All data are presented as mean  ±  SD.

^a^P value paired sample t-test

^b^P value independent sample t-test

*^c^P value as an implication to compare the mean changes of each parameter between the two groups before and after supplementation (ANCOVA), adjusted for the primary variable, changes in energy intake, BMI, WHR and MET

*Abbreviations* WC: waist circumference; HC: hip circumference; BMI: body mass index; WHR: waist to hip ratio; SBP: systolic blood pressure; DBP: diastolic blood pressure; HDL: high density lipoprotein; LDL: low density lipoprotein; HOMA-IR: homeostasis model assessment of insulin resistance; QUICKI: Quantitative Insulin Check Index

The consumption of PUFA w-6 (*P* = 0.028) and vitamin E (*P* = 0.029), was significantly decreased at the end of week 12 in subjects assigned to soy isoflavone group compared to baseline. According to Table 3, there were no significant changes in the intake of total energy and macronutrients. In addition, no significant changes observed regarding other dietary variables and physical activity within and between the both groups (*P* > 0.05). (Data not shown)

**Table 3:** Mean changes (SD) from baseline in calories and macronutrients by treatment groups

variables	Groups	Baseline	After 12 weeks	Changes (%)	*P* value ^a^
Total Energy intake (kcal/day)	Soy isoflavone	2082 ± 707.19	1981 ± 538.58	­100.85 ± 783.37	0.526
	Placebo	2111 ± 694.58	1985 ± 1108.32	­126.50 ± 1235.43	0.644
	P value ^b^	0.890	0.990	0.932	
Total carbohydrates (percent of kcal/day)	Soy isoflavone	43.81 ± 9.31	47.81 ± 6.84	3.99 ± 11.52	0.096
	Placebo	70.52 ± 97.34	92.46 ± 141.05	21.94 ± 178.72	0.580
	P value ^b^	0.179	0.120	0.618	
Total protein (percent of kcal/day)	Soy isoflavone	14.59 ± 3.38	15.04 ± 2.04	0.45 ± 3.06	0.465
	Placebo	83.93 ± 318.94	162.81 ± 468.55	78.88 ± 585.48	0.544
	P value ^b^	0.282	0.121	0.506	
Total fat (percent of kcal/day)	Soy isoflavone	43.52 ± 8.26	39.42 ± 5.32	-4.09 ± 9.74	0.067
	Placebo	288.26 ± 1143.25	191.56 ± 477.80	-96.69 ± 1269.79	0.375
	P value ^b^	0.289	0.118	0.717	

All the variables demonstrated as mean ±  SD

^a^P value attained from paired sample t-test

^b^P value based on independent sample t-test

Macronutrients are reported as the percent of total calories intake

## Discussion

In the present study the effects of soy isoflavones in patients with NAFLD are evaluated. According to findings of the present study, supplementation with 100 mg/d soy isoflavones for 12 weeks improved lipid profile and reduced waist and hip circumferences along with life style modification in patients with NAFLD. There are several studies indicating the positive effects of soy isoflavones on lipid profile. Based on a study by Wang et al. [39] higher consumption of total isoflavones, daidzein, genistein and glycitein is inversely associated with hyperlipidemia in NAFLD patients. Based on findings from some former randomized clinical trials (RCTs), supplementation with 70 mg/day isoflavone for 12 weeks, declined the level of serum TG and isoflavone supplementation of 61.8 mg/day for 4 weeks had favorable effects on serum lipid levels [40, 41]. Moradi et al. have shown the effect of isoflavones in lowering the level of serum total cholesterol and apoprotein B in post-menopausal women in a recent meta-analysis of RCTs [42]. In a review article, Xia et al. have also reported the hypolipidemic effects of soy ingredients in both animal and human studies [43]. the underlying mechanisms by which, soy isoflavones may insert their hypolipidemic effects are; the first, similarity between soy isoflavones and 17-β-esteradiol that making soy isoflavone as a ligand for estrogen receptors (ERs) which, resulted in gene expression and advantageous effects on lipid profile [44, 45]. The second, soy isoflavone’s hypolipidemic effects can proceed in ER-independent pathway. Soy isoflavones can induce the gene expression of PPARα and trigger the activation of adenosine 5′-monophosphate (AMP)-activated protein kinase (AMPK). Altogether, they increase the induction of genes involved in lipoprotein metabolism. Thus, the production of particles rich in TG decreases and their breakdown increases. In addition, fatty acids tend to be more utilized and go under catabolic processes [46–48]. Moreover, soy isoflavone acts as an obstacle for expression of genes involved in de novo lipogenesis such as sterol regulatory element binding protein-1c (SREBP-1c) and carbohydrate regulatory element binding protein-1 (ChREBP) [49, 50]. Other possible explanation in regard to isoflavone’s lipid lowering effect is their role on gene expression of enzymes participating in lipid conversion processes named lipoprotein lipase (LPL), hepatic lipase (HL), also known as hepatic triglyceride lipase (HTGL) and 7alpha-hydroxylase [51, 52]. In our study, soy isoflavone intake had no effect on the level of HDL-C in NAFLD patients. In line with this finding, several previous studies have revealed that isoflavone intake makes no changes to serum HDL level. Zhang et al. [53] have reported that soy isoflavone supplementation has significant decreasing effects on TC and LDL but no significant effects on serum HDL. According to findings of a recent meta-analysis of RCTs, there were no significant changes in regard to HDL level after soy isoflavone supplementation [54]. A brief report has also indicated the same result in terms of soy isoflavone effect on serum HDL [55]. In other words, based on findings of the present study, a non-significant increase of HDL level in soy isoflavone group observed which, is in line with the majority of former studies’ findings [56–58]. Unlike our finding, several studies have implied the increasing effects of soy isoflavone on HDL-C [17, 59–61]. This discrepancy may be explained by the supplementation duration, the dosage of soy isoflavone and baseline value of HDL which, in the present study was near normal range. This study demonstrated no significant effect of soy isoflavone intake on glycemic parameters. This finding is in agreement with several previous research [55, 62–64]. Moreover, the same results have been yielded according to several meta-analysis of RCTs. Bara´nska el tal. have reported no significant effect of soy protein or/ and isoflavones on serum glucose level and HOMA-IR [54]. Yang et al. also reported that soy protein or/ and isoflavones has no significant impact on the serum level of fasting blood glucose [65]. However, there are some studies with contradictory results implying the positive effect of soy isoflavones on glycemic control [21, 66–69]. The reasons behind these inconsistent results may be due to the fact that in the present study the baseline level of serum fasting glucose and insulin was normal in subjects of soy isoflavone group. It seems that, the better results observed in case of hyperglycemia and diabetes. Thus, it is sensible to find no significant improvement on glycemic parameters in this group. On the other hand, in placebo group, the baseline level of serum glucose was significantly higher than that of the soy isoflavone group and as one’s expected, the level of serum glucose was significantly decreased only in placebo group. Also, the reduction of HOMA-IR in this group of patients was close to the significant level. Furthermore, according to findings of the current research, no significant impact of soy isoflavone observed on blood pressure including both SBP and DBP in patients with NAFLD. Based on a meta-analysis of RCTs by Liu et al. [70] soy isoflavone can reduce the blood pressure only in hypertensive patients but not in normotensive subjects. In the present study, the mean baseline value of SBP and DBP was normal in both groups which, could explain the non-significant effect of soy isoflavone on blood pressure in this investigation. Because the normotensive people have high flow mediated dilation and normal endothelial function [70]. Another meta-analysis of RCTs have reported that soy isoflavone intake causes no changes to SBP and DBP based on four included RCTs. After subgroup analyses, it was demonstrated that supplementation with soy isoflavone for more than 6 months has led to significant decline in SBP and DBP [71]. In our research, the duration of soy isoflavone supplementation was shorter than that of mentioned so, this could be another reason for this null observation. Additionally, soy isoflavone intake resulted in reducing WC within both groups and HC in soy isoflavone group after 12 weeks of supplementation. Amanat et al. has reported the same results in regard to WC decrement in genistein treated group but changes to other anthropometric parameters remained insignificant [66]. The underlying mechanisms explained by inducing effect of soy isoflavone on genes involved in fatty acid catabolism such as PPARα, AMPK and very long-chain acyl -CoA dehydrogenase in conjunction with suppressive effects on genes in charge of lipogenesis like SREBP-1c, PPARγ and acetyl-CoA carboxylase 2 in animal models [72–74]. Totally, there are several recent studies investigating the effects of soy or soy isoflavones on metabolic syndrome, which have yielded with the positive results [75–77]. Overall, in this study soy isoflavones have led to improve MAFLD by positive effects on some markers of metabolic syndrome. This result is in line with the animal study, which reported that genistein was effective in MAFLD patients [78].

Conducting fibroscan to measure fibrosis which, is more accurate than sonography used in the majority of the previous studies and recruiting newly diagnosed NAFLD patients before going under serious medication treatments are several strengths have been attributed to the present study. However, small sample size and unmeasured serum level of soy isoflavones are some of our study’s limitations which are necessary to consider.

## Conclusions

In conclusion, daily administration of 100 mg/day soy isoflavones can reduce serum concentration of triglyceride, LDL and total cholesterol and resulted in waist and hip circumferences decrement as markers of metabolic status in NAFLD patients.

## Data Availability

Raw data that support the findings of this study are available from the corresponding author, upon reasonable request. The email address of the corresponding author: bizhanhelli@yahoo.com.

## Abbreviations

AMPK: Adenosine 5′-Monophosphate -activated Protein Kinase
ANCOVA: Analysis of Covariance
BMI: Body Mass Index
ChREBP: Carbohydrate Regulatory Element Binding Protein-1
DBP: Diastolic Blood Pressure
FBS: Fasting Blood Glucose
HC: Hip Circumference
HDL: High Density Lipoprotein
HL: Hepatic Lipase
HTGL: Hepatic Triglyceride Lipase
HOMA-IR: Homeostatic Model Assessment-Insulin Resistance
LDL: Low Density Lipoprotein
LPL: Lipoprotein Lipase
MET.h.d: Metabolic Equivalent Hours per day
MAFLD: Metabolic associated fatty liver disease
NAFLD: Non-Alcoholic Fatty Liver Disease
NO: Nitric Oxide
PPARs: Peroxisome Proliferator Activated Receptors
QUICKI: Quantitative Insulin Check Index
RCTs: Randomized Clinical Trials
SBP: Systolic Blood Pressure
SD: Standard Deviation
SPSS: Statistical Package Software for Social Science
SREBP-1c: Sterol Regulatory Element Binding Protein-1c
TC: Total Cholesterol
TG: Triglyceride
THHC: Taleghani Hospital Hepatology Clinic
WC: Waist Circumference
WHR: Waist To Hip Ratio
